# Removal of a Large Encephaloid Tumor

**Published:** 1848-06

**Authors:** W. L. Sutton

**Affiliations:** Georgetown, Ky.


Art. II.—*Removal of a large Encephaloid Tumor.* By W. L. Sutton, M.D., of Georgetown, Ky.

On the 24th of April, 1846, I was desired to visit a negro woman aged 50, belonging to Dr. W. G. Offutt, of Woodford county. I found, occupying the right mamma, a large, exceedingly painful tumor, soft to the touch, and giving a sensation strongly resembling that of fluctuation—so strongly indeed, as to have induced a neighboring physician to introduce a lancet to discharge pus. None, however, was discharged. Dr. O. himself, on the day preceding my visit, had punctured the tumor in two places with the same result. Subsequently to the last punctures, a small quantity of pus was discharged from the one first made. The surface of the tumor upon the top, and for a considerable distance down the sides, was smooth and of a dark red color; approaching the chest this color gradually changed to that of the natural color of the skin. The dimensions of the tumor were as follows:

| | |
|---|---|
| Circumference at the base, | $19\frac{1}{4}$ inches |
| " 3 inches from the base, | $17\frac{1}{8}$ " |
| Measure diagonally over the tumor in the direction of the lower edge of the pectoralis major | $14\frac{3}{8}$ " |
| Measure over its smallest bulk | 11 " |

From the orifice of the first puncture, a fungus was protruding about the size of a partridge-egg, of a color resembling brain with many red papillæ on the surface. The woman was much emaciated; had not been able to sleep for several months on account of the excruciating pains in the tumor; pulse 110 and irritable; no appetite, but the bowels in tolerable order.

I had seen this breast once, about 18 months previously, when the tumor was much smaller, had considerable firmness —no approximation towards a sensationof fluctuation—was not discolored or painful.

I directed the tumor to be suspended as much as possible; the patient to take at night, calomel grs. 15 with a fourth of a grain of sulph. morphia. The same dose to be repeated on the night of the 27th; the calomel to be purged off on the succeeding day with rhubarb if necessary. A fourth of a grain of sulph. morphia to be given each intervening night.

29th. Visited her again; found her decidedly more comfortable. She had been able to get some quiet sleep under the influence of the morphia: pulse had come down to about 100, and of a more quiet character. The first incision now presented a fungus with a base somewhat larger than the transverse section of a hen's egg. A fungus of a similar character now protruded from each of the other punctures.

Although there was little to promise ultimate success, we determined to remove the tumor. Having administered two grains and a half of opium half an hour previously, we (Dr. George C. Sutton and myself) proceeded to the operation. Two elliptical incisions were made parallel to the long diameter of the base, including all that portion of the skin which we considered important to remove. The base of the tumor was then dissected from the walls of the thorax. About a pint of blood was lost: three arteries required the ligature. Upon laying down the flaps, we found a surface, 1½ inches by 4, uncovered by integuments. This was dressed with lint spread with cerate. A fourth of a grain of sulph. morphia was directed to be taken every night at bed time; small doses of calomel and rhubarb every third night.

May 7th. The flaps have united by the first intention: there is nothing to indicate the location of the ligatures (which were of catgut, and cut off close to the knot.) A considerable portion of the lower end of the raw surface presents a rather pallid fungous appearance. Otherwise the appearance is good: pulse 90, sleeps tolerably well, appetite improved—is cheered with the prospect of recovery. ℞. solut. sulph. cupri to the fungous surface. Continue the morphia at night; cal. and rhubarb occasionally; attention to diet and bowels.

29th. The surface healed over, except a space an inch long by half an inch wide; this looks very well. General health very much improved. From this time she steadily improved: the surface soon skinned over, and she is now, April 1848, in good health.

Upon laying open the tumor after its removal, it was found to consist of a soft mass very much of the consistence (perhaps less firm) and color of brain: around this was a slight, condensed substance, this might have been considered a cyst, or a condensation of cellular membrane, according to the fancy of the observer.

Remarks.—I think there is little reason to doubt that this was a genuine encephaloid tumor. If so, the result has been more satisfactory than I was prepared to expect, and consequently the prognosis of such tumors as at present made, *may be* too gloomy. Upon a careful examination of the case before the operation, I was of opinion, that without the removal, the patient would live but a short time—that with it, her life might be prolonged a few months, but that diseased action would soon reappear and carry her off.

April, 1848.

